# Colocaval Fistula: A Unique Case Report

**DOI:** 10.1155/cris/8818123

**Published:** 2025-07-29

**Authors:** Stephen Vining, Brett M. Chapman

**Affiliations:** ^1^School of Medicine, LSU Health Shreveport, Shreveport, Louisiana, USA; ^2^Department of Surgery, LSU Health Shreveport, Shreveport, Louisiana, USA

**Keywords:** case report, colocaval, enterocaval, fistula

## Abstract

Fistula formation is a connection between anatomic locations that is intrinsically abnormal. A variety of causative etiologies and involved structures exist for these anomalous developments. Fistulas between vasculature and the enteric system are rare. When present, anatomical proximity is the dominant factor in determining which structures are involved. Aortoenteric fistulas involving the esophagus, duodenum, and small bowel are well-known with the stomach also being involved in rare instances. Fistulas involving the inferior vena cava (IVC) and enteric system have also been seen with the stomach, small bowel, and intrathoracic colon following an interposition each represented in reported cases. We present a case of an 82-year-old female with multiple medical comorbidities including opioid dependence, chronic constipation, recurrent lower extremity deep venous thrombosis, recurrent upper gastrointestinal (GI) bleeding, and IVC filter dependence who developed a unique problem. Her presenting complaints were nonspecific, but ultimately a diagnosis of fistula formation between the IVC and sigmoid colon was made. The colocaval fistula described here is the first intraperitoneal case to be reported in the body of literature.

## 1. Introduction

A fistula is a connection between two body parts that is anatomically abnormal. The word fistula comes from the Latin *fistula* meaning “pipe, tube” and first appearing in the late 14th century. Fistulas can form due to injury, surgery, inflammation, infection, radiation, foreign body, or pressure in proximity. A variety of structures including arteries and veins, trachea, esophagus, bowel, gallbladder, bile duct, bladder, vagina, uterus, skin, and more can be involved.

Fistula formation between the inferior vena cava (IVC) and gastrointestinal (GI) tract, however, is a rare phenomenon. Due to anatomical proximity, the majority of reported cases involve the IVC and duodenum. Less commonly, gastrocaval or jejunocaval fistulas have been encountered [1]. Enterocaval fistula (ECF) formation has occurred following foreign body ingestion, penetrating trauma, peptic ulcer disease, malignancy, radiotherapy, and IVC filter migration [2]. Diagnosis is often a challenging endeavor and requires multiple modalities, as patients can present with nonspecific symptoms such as fever and altered mental status [3]. Treatment has historically involved open surgery with a variety of resection or reconstruction techniques for both the IVC and the involved component of the GI tract. ECF represents a medical emergency with a mortality rate as high as 33% [2].

While ECF is rare in totality, our description of an intraperitoneal colocaval fistula is, to our knowledge, the first such case to be reported in the literature.

## 2. Case Report

An 82-year-old African American female with a complex medical history specifically notable for long-standing opioid dependence, chronic constipation, recurrent lower extremity deep venous thrombosis, pulmonary embolus, recurrent lower GI bleed of unidentified location, and IVC filter placement 7 years prior presented to an academic tertiary referral center via emergency medical services with complaints of fatigue, confusion, and mild abdominal discomfort for 2 days. Her daughter, who served as her medical power of attorney and healthcare proxy, was present as well and participated in the encounter.

**Figure 1:**
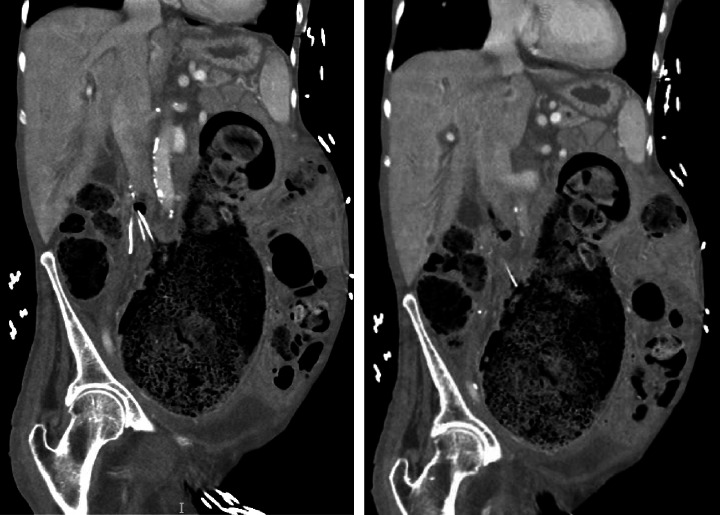
CT abdomen and pelvis coronal views. Large, dilated, and stool-filled sigmoid colon. Malpositioned IVC filter leg (long strut) penetrating the colon wall with associated intracaval air at the filter neck visible laterally.

**Figure 2:**
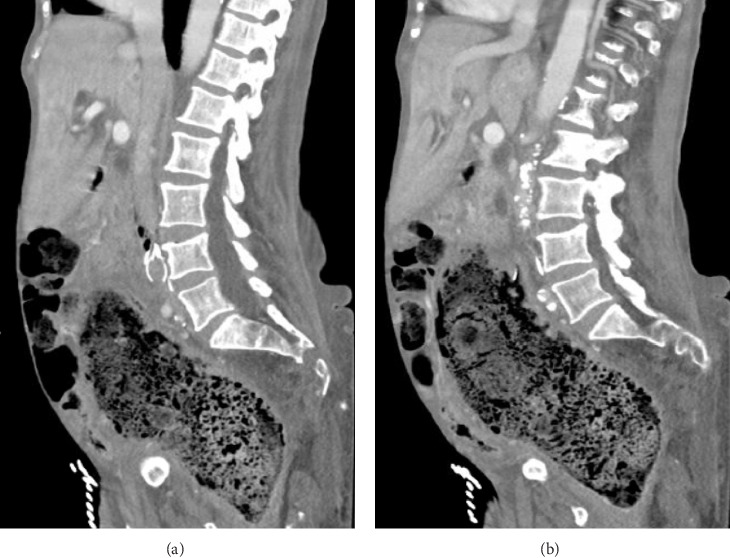
CT abdomen and pelvis sagittal views. Intracaval air cranial to filter arms (a). Filter leg in colon wall (b).

**Figure 3:**
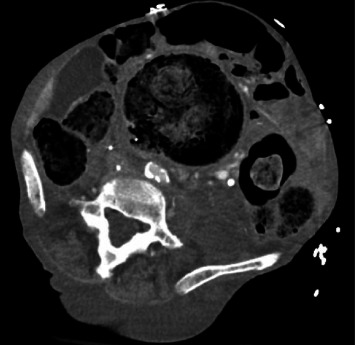
CT abdomen and pelvis axial view. Three filter legs visible, including image right (patient left) where the filter leg is within the colon lumen.

She was awake and alert with mild apparent discomfort on initial evaluation. She was disoriented to time, but oriented to person, place, and situation. She was febrile to 101.8° Fahrenheit, tachycardic to 118 beats per min, borderline hypotensive with a systolic blood pressure of 108 mmHg, and tachypneic to 22 breaths per min. Her oxygen saturation was 98% on room air. A thorough review of systems was notable for subjective fever and chills, suprapubic abdominal pain, nausea, dysuria, and urinary frequency as well as notably negative for bleeding per rectum. The physical exam was remarkable only for a palpable fullness in the left lower quadrant with no significant distention or tenderness and no peritonitis. Laboratory studies revealed leukocytosis (WBC 23.7 K/µL) with no left shift (Gran 65.0%) and 18% bandemia, hypokalemia (K 3.4 mmol/L), azotemia (BUN 28 mg/dL), elevated alkaline phosphatase (ALP 163 U/L), protein calorie malnutrition (Alb 2.0 g/dL), transaminitis (AST 63 U/L, ALT 22 U/L), troponinemia (Trop 0.059 ng/mL), and lactic acidosis (Lac 4.8 mmol/L). Urinalysis was unrevealing. Blood cultures were drawn with polymicrobial growth of *Escherichia coli*, *Pseudomonas aeruginosa*, and *Paraclostridium bifermentans* within 8 h of collection. Cross-sectional imaging was obtained with a contrast-enhanced computed tomography (CT) scan. Imaging revealed a massively dilated rectum and sigmoid colon measuring 10.4 cm in maximum diameter with a large volume of impacted stool. The indwelling IVC filter was malpositioned with a rightward tilt and a filter leg (long strut) penetrating the walls of the IVC and colon. Additionally, there was a sizeable focus of intracaval air located at the junction of the filter hook and cranial aspect of the filter arms (short struts) and legs (Figures 1–3).

Colorectal surgery, vascular surgery, and critical care medicine teams were consulted by the emergency department providers. Treatment for bacteremia and sepsis was started with crystalloid volume expansion and broad-spectrum antibiotics. Complex multidisciplinary discussions were had among the consultants and the patient and family. The patient was offered hospital admission for antibiotics, resuscitation, and management of her sepsis. The patient and family were informed that any potential intervention would be high-risk and carry significant morbidity and potential for mortality. Discussions included preliminary and less invasive options such as manual and medication-assisted disimpaction with lower endoscopy and venography to follow. More definitive surgical options were also discussed and likely to include venotomy, removal of the IVC filter, caval repair versus reconstruction, primary repair of the colon versus partial colectomy with likely fecal diversion. The patient was not interested in any aggressive treatment plans. She expressed her desire to arrange hospice and return home. Home hospice arrangements were made and she expired shortly after leaving the facility 13 h after her initial presentation.

## 3. Discussion

ECF formation is a rare event with a total of 60 reported cases. Duodenocaval fistulas (DCF) account for 55 of 60 (91.7%) ECF cases. The most common etiology of DCF formation is IVC filter migration [2, 4–6]. IVC filters have been used for pulmonary embolus prevention since the first filter device was developed by Mobin-Uddin et al. [7] while on faculty at the University of Miami. He published his findings in the Archives of Surgery and New England Journal of Medicine in 1969 and 1972, respectively. Newsweek magazine reported on the milestone in its October 20, 1969, issue calling it the “umbrella of life.” The Mobin-Uddin IVC filter was released for general clinical use in 1970, but was soon replaced by the more popular Greenfield filter when it came to market in 1973 [7–9]. Multiple IVC filter options in both permanent and retrievable manufacturer designs are now available, including the Cook Celect Platinum Vena Cava Filter that was placed in our patient via a right internal jugular vein approach 7 years prior to presentation. The introduction of retrievable IVC filters and expansion of relative indications for filter placement has led to an increase in the number of procedures to more than 65,000 per year, but filters are not without inherent risk [10, 11]. IVC perforation rates of up to 70% have been reported depending on the type of filter [12]. A review by Negmadianov et al. [13] documented 49 cases of IVC perforation with accompanying duodenal involvement. The inherent risk of device-related IVC penetration is reflected as the leading case of DCF formation. Some other described etiologies in order of incidence include abdominal radiotherapy, peptic ulcer disease, and foreign body ingestion [2, 5, 14, 15]. ECF formation involving sites in the GI tract other than the duodenum are extremely rare with only five such cases previously described (Table 1). Gastrocaval fistula formation has been reported on three occasions with two being sequelae of peptic ulcer disease and one the result of penetrating trauma [2, 16, 17]. Jejunocaval fistula formation has been described once following resection of a gastroesophageal junction adenocarcinoma and creation of an esophagojejunal anastomosis [18]. Colocaval fisulta formation has been described on one occasion following an esophagectomy and colon interposition enteric reconstruction [19]. To the best of our knowledge, this is the only case of intraperitoneal colocaval fistula to be reported to date. The only reported case even similar in nature occurred following neoadjuvant radiation for rectal adenocarcinoma with a resultant rectal perforation and large chronic intra-abdominal abscess formation that subsequently fistulized to the IVC in a delayed manner [20].

Diagnosis of ECF is often a challenge, as patients commonly present with nonspecific complaints, highlighting the need for the requisite high index of suspicion. GI bleeding is the most common presenting sign with fever being next most likely. However, both GI bleeding and fever occurring simultaneously has been found in only one-third of patients. Other common symptoms that have been noted on presentation are abdominal pain, nausea, vomiting, and confusion [21]. Blood culture growth is often polymicrobial, and fungemia is not uncommon [21, 22]. CT scan alone can be an effective tool in the diagnosis of ECF, especially in cases involving an ingested foreign body or migrated IVC filter [23]. Besides obvious foreign bodies, CT is capable of visualizing air or thrombus in the IVC and abscesses in the area of the involved bowel [22]. Esophagogastroduodenoscopy (EGD) is another technique that has been successfully utilized in the diagnosis of ECF. This is especially true of cases involving an IVC filter, where the filter strut is visibly penetrating the intestinal wall [13]. Colonoscopy has not been previously described as an investigative or therapeutic modality given the lack of prior ECF involving the normal anatomic colon in the existing literature. Lower endoscopy could be considered in an appropriate case, but we would caution its use given the concern for colonic motion during the procedure and potential detrimental sequelae. Other less common modalities that have been utilized to successfully diagnose ECF include magnetic resonance imaging (MRI), cavography, and upper GI series [22, 24]. None of these diagnostic modalities are foolproof, however, with incidental ECF diagnosis at the time of abdominal surgery or autopsy in 29% and 23%, respectively [2].

ECFs are likely to be lethal without intervention. Generally, interventional techniques have revolved around laparotomy, direct repair of the intestinal defect, and venorrhaphy [5, 15]. In instances involving an IVC filter, venotomy with direct removal of the filter has commonly been employed. Segmental resection of the involved portion of the IVC has been performed in cases involving a filter with accompanying thrombus [13]. While treatment has usually involved open surgery, there are exceptions. EGD with endoscopic intervention alone has been successfully utilized in the treatment of a DCF secondary to an ingested foreign body [25]. Endovascular intervention is a consideration in select cases, but is often not an option in the setting of incorporated and malpositioned IVC filter devices that carry high risk of caval injury during a retrieval attempt. Endovascular intervention has been successfully utilized for temporary stabilization of a DCF in a poor surgical candidate [3]. IVC filters in general have been retrieved using endovascular, laparoscopic, and robotic techniques [26]. The latter two techniques have yet to be employed directly in the treatment of an ECF.

Our patient presented with a sigmoid colocaval fistula, a unique subtype of ECF due both to the general rarity of the pathology and the lack of normal anatomical proximity. Her nonspecific symptoms of altered mental status, fatigue, and vague abdominal discomfort are common among observed cases. Her lack of GI bleeding was likely due to the significant degree of fecal impaction. She was counseled that disimpaction could precipitate a fatal lower GI hemorrhage. Blood cultures rapidly returned polymicrobial bacteremia with both Gram-positive and Gram-negative rods. CT scan alone was sufficient to diagnose her condition by showing the malpositioned IVC filter within the colon lumen and confirmatory sign of intracaval air. A review of prior cross-sectional imaging from approximately 5 years prior to this presentation and 18 months after initial filter placement revealed filter migration distally by an estimated 22 mm in additional to the rightward tilt. Had she elected to pursue treatment with curative intent, care would have centered on control of her bacteremia and sepsis to optimize her peri-operative morbidity and mortality with a complex operation including venotomy, removal of IVC filter, IVC repair versus reconstruction, and colon repair versus resection to follow.

## 4. Conclusion

Given its infrequent incidence, suspicion for ECF should likely remain low in most instances. However, as our present report demonstrates, the potential for ECF should never be totally excluded. This is particularly true in those patients who present with vague symptoms and have a recognized risk factor such as an IVC filter.

## Figures and Tables

**Table 1:** Etiology, presentation, and diagnostic modality of nonduodenocaval enterocaval fistulae.

Type of fistula	Total no.	Etiology of fistula	Presentation	Diagnostic modality
Gastrocaval [2, 16, 17]	3	Peptic ulcer disease (2) trauma	Recurrent melena, dysphagia Pain, vomiting, pneumoperitoneum Stab wound, hematemesis	EGD Laparotomy CT
Jejunocaval [18]	1	Esophagojejunal anastomosis	Sepsis	CT
Colocaval [19]	1	Esophageal interposition	Sepsis, hematemesis	Thoracotomy

## Data Availability

There is no relevant data outside what is included in the manuscript.

## Supporting Information

Supporting Information SCARE checklist.
